# Atrial Fibrillation in β-Thalassemia: Overview of Mechanism, Significance and Clinical Management

**DOI:** 10.3390/biology11010148

**Published:** 2022-01-17

**Authors:** Michele Malagù, Federico Marchini, Alessio Fiorio, Paolo Sirugo, Stefano Clò, Elisa Mari, Maria Rita Gamberini, Claudio Rapezzi, Matteo Bertini

**Affiliations:** 1 Cardiology Unit, Azienda Ospedaliero-Universitaria di Ferrara, 44124 Ferrara, Italy; federico.marchini@edu.unife.it (F.M.); alessio.fiorio@edu.unife.it (A.F.); paolo.sirugo@edu.unife.it (P.S.); stefano.clo@edu.unife.it (S.C.); claudio.rapezzi@unife.it (C.R.); matteo.bertini@unife.it (M.B.); 2 Day Hospital Thalassemia and Hemoglobinopathies, Azienda Ospedaliero-Universitaria di Ferrara, 44124 Ferrara, Italy; e.mari@ospfe.it (E.M.); m.gamberini@ospfe.it (M.R.G.)

**Keywords:** thalassemia, hemoglobinopathy, arrhythmias, supraventricular, atrial fibrillation, iron, ablation, chelation, heart

## Abstract

**Simple Summary:**

Atrial fibrillation in patients with β-thalassemia has a higher incidence compared to the general population. Its pathophysiology is peculiar and strictly related to anemia, iron overload, hemolysis, inflammation, atrial dilatation, fibrosis, and remodeling. The clinical presentation is that of a highly symptomatic disease with frequent recurrencies, significantly impacting over quality of life and prognosis. Furthermore, the risk of thromboembolic events and stroke is considerable. The available treatments are drug therapy and transcatheter ablation. Moreover, particular attention should be paid to anticoagulant therapy for thromboembolic prophylaxis.

**Abstract:**

Thalassemia is an inherited blood disorder with worldwide distribution. Transfusion and chelation therapy have radically improved the prognosis of β-thalassemic patients in the developed world, but this has led to the development of new chronic cardiac complications like atrial fibrillation (AF). Prevalence of AF in patients with β-thalassemia is higher than in the general population, ranging from 2 to 33%. Studies are lacking, and the little evidence available comes from a small number of observational studies. The pathophysiology is not well understood but, while iron overload seems to be the principal mechanism, AF could develop even in the absence of iron deposition. Furthermore, the clinical presentation is mainly paroxysmal, and patients are highly symptomatic. The underlying disease, the pathophysiology, and the clinical presentation require a different management of AF in β-thalassemia than in the general population. Rhythm control should be preferred over rate control, and the most important antiarrhythmic therapy is represented by chelation drugs. Thromboembolic risk is high, but the available risk scores are not validated in β-thalassemia, and the choice of anticoagulation therapy should be considered early. The main purpose of this review is to summarize the actual knowledge about AF in β-thalassemia, with a specific focus on the clinical management of these complex patients.

## 1. Introduction

Since the introduction of transfusion and chelation therapy, the natural history of β-thalassemia has changed. While for millennia, patients died in childhood, and 50 years ago, following the introduction of transfusion therapy, they died young of severe heart failure and hemochromatosis secondary to iron overload, at the present time, with optimal care, most thalassemia patients have a longer life expectancy and survive into their 60s or later. Today, an emerging problem in the natural history of thalassemia is the development of atrial fibrillation (AF). The mechanisms leading to AF in thalassemic patients are different from those in the general population, and the clinical features are peculiar. What is the meaning of this arrhythmia? Many points are still obscure, and specific studies are few, but the management of thalassemic patients with AF has entered daily clinical practice. This review aims to analyze the various aspects of the problem and to explore possible management strategies based on the available evidence.

## 2. Epidemiology

### 2.1. Thalassemia

Thalassemia is the most common inherited disease. Incidence and prevalence are not uniform around the world, since they have a specific distribution which is higher in a geographic area extending from the regions of Southeast Asia through Middle East to the Mediterranean Sea [1]. The reasons for this peculiar epidemiology have both cultural backgrounds, like the high prevalence of consanguineous marriages, and pathophysiological explanations. In fact, being β-thalassemia gene carriers protects against mortality from malaria infection [2,3,4].

In 2008, a report from the World Health Organization estimated an incidence of β-thalassemia of 40,000 newborns each year and a total annual incidence of symptomatic patients of 1/100,000 people around the world and 1/10,000 people in Europe [5,6].

Prevalence is high, with gene carriers representing 1.5% of the global general population (approximately 90 million people), of whom the great majority are in developing countries [3]. In recent years, prevalence in Europe and in North America has been increasing because of migration, and major hemoglobinopathies are now the most common genetic diseases in Europe [2,6,7,8].

The epidemiology of β-thalassemia is changing. Migration, but also the more recent movements of refugees and the major adoption rates of children from conflict areas, have increased the prevalence of the disease in regions with a previously low prevalence, while, at the same time, prevention and screening programs in endemic regions have reduced the number of affected individuals [1,2,9,10].

Mortality remains high. In a retrospective cohort analysis of British β-thalassemic patients, the 10-year mortality rate from 2009 to 2018 was 6.2%, which means that it was more than five times greater than the age-/sex-adjusted mortality of the general population [11]. Another retrospective cohort study of β-thalassemic patients in Iran showed 20-, 40-, and 60-year survival rates of 85%, 63%, and 54%, respectively [2,12].

Transfusion and chelation therapy have radically improved the prognosis of β-thalassemic patients in developed countries, where 80% of patients survive to over 40 years of age. The higher survival rate is mainly due to the reduction in cardiac-related mortality, even if cardiovascular diseases remain the most common cause of death [13,14,15]. In developing countries, like those in the Middle East, screening programs have reduced the prevalence of the disease, but there are still regions where such tools have been denied because of limitations in the local public health agenda or cultural and religious beliefs [1,2]. Finally, survival remains low in countries where patients have no access to transfusion or iron chelation therapy.

The increase in survival has led to a high rate of new comorbidities: the previously cited British cohort showed that, while in the 10–14 age range, 60% of patients suffered from one or more comorbidities, this value rises to 95% after the age of 50. The most common diseases were endocrine disorders (40%), osteoporosis (40%), and diabetes (34%). Cardiac diseases affected 18% of patients, with the most common conditions being AF (11%), heart failure (9%), and acute arrhythmias (4%) [11].

### 2.2. Atrial Fibrillation in Thalassemia

AF typically affects 2–4% of the general population, but in patients affected by β-thalassemia, the prevalence is definitely higher, ranging from 2 to 33% [15,16,17,18]. Notably, studies reporting AF prevalence in β-thalassemia are heterogeneous, and the studied populations differ in characteristics like age, iron overload, and chelation regimens, since the epidemiology of AF was not the main purpose of these studies [15]. For example, studies conducted on the Myocardial Iron Overload in Thalassemia (MIOT) database showed a low prevalence of AF in β-thalassemic patients, ranging from 2.6 to 3.2% [19,20]. In other studies, instead, like those of Kirk or Kostopoulou, AF was present in 12% and 26% of patients, respectively [21,22]. A cross-sectional analysis from a small British cohort reported the highest prevalence of 33.8% [17].

### 2.3. Thromboembolism

Identifying patients at high risk of AF is particularly important, since β-thalassemia is associated with an increased risk of thromboembolic events due to a chronic hypercoagulability state. In the work of Taher at al., the clinically relevant prevalence of thromboembolic events was 1.65%, with a greater risk for thalassemia intermedia than thalassemia major (3.9 vs. 0.9%). Risk factors for thrombosis were age > 20 years, previous thromboembolic events, family history, and splenectomy. 

Among thromboembolic events, ischemic stroke, in particular, has been reported in 0.25–0.46% of patients with β-thalassemia [23,24]. No specific studies have investigated the characteristics or the treatment of ischemic stroke in β-thalassemic patients. However, in a small case series, the occurrence of large hemispheric stroke in patients with β-thalassemia was associated with the presence of AF and cardiac iron overload, leading the authors to hypothesize an underlying cardiogenic embolism rather than a hypercoagulability-associated mechanism and suggesting a possible role for preventive anticoagulant therapy [25].

## 3. Pathophysiology

**Figure 1:**
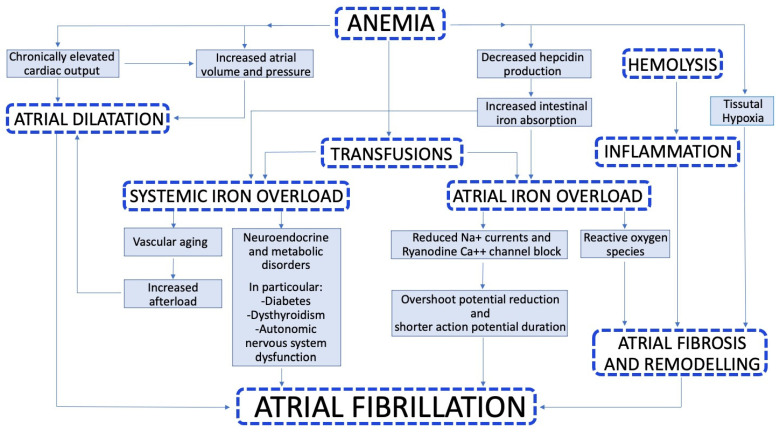
Pathophysiology of atrial fibrillation in β-thalassemia.

The pathophysiology of AF in β-Thalassemia is a complex mechanism involving iron overload, anemia, coexisting metabolic and endocrine disorders, and accelerated vascular aging (Figure 1) [15,26].

The need for frequent blood transfusions leads to iron overload and, once the capacity for iron storage is exceeded, labile forms of iron enter the circulation and can infiltrate into cardiomyocytes [27,28]. Animal experiments indicated that iron toxicity affects the electrical conduction in the heart and may lead to arrhythmias. In addition, free iron can potentially be dangerous due to intracellular reaction, in which ferrous iron is oxidized by hydrogen peroxide to ferric iron, generating highly reactive hydroxyl radicals. Hydroxyl radicals are the most reactive oxygen species (ROS) and mediate severe damage to crucial cellular components, especially in those cells that use a large amount of oxygen, like cardiomyocytes [29]. Moreover, significant increases in ROS have deleterious effects on cellular pathways and disrupt crucial biological reactions and processes, which may lead to many pathological conditions including arrhythmia and AF [30].

Iron overloaded cardiomyocytes have a smaller overshoot potential and shorter action potential duration than iron-free cardiomyocytes [31]. The reduced overshoot potential results in a decreased depolarization (phase 0 of the action potential, fast Na^+^ current). The reduced late fast Na^+^ current during the plateau phase may determine a shortening of the action potential duration because of an imbalance between the small currents. Furthermore, iron overload is involved in block of ryanodine Ca^++^ channels and in changes of sarcoplasmic Ca^++^ release and reuptake mediated by oxidative stress. All these factors result in electrical heterogeneity, which is the substrate for triggered activity and re-entry circuits, representing the mechanism of origin of arrhythmias [32].

There is clear evidence supporting the association between iron deposition and incidence of arrhythmias. In a prospective, multicenter observational study, patients with severe iron overload, defined as T2* < 6 ms detected by cardiac MRI at interventricular septum, had a higher incidence of arrhythmic events in 1 year of follow-up (relative risk 8.79, 95% CI 4.03–19.2) and AF was the most frequent arrhythmia [21]. However, arrhythmias were also present even in the absence of iron overload detectable at MRI. This finding is very interesting, and indicates that the atrial function may be impaired before the iron deposition becomes evident. Many possible explanations can be hypothesized. On one hand, the hyperkinetic flow of these anemic patients may alter early the atrial function and the atrium itself may undergo structural alterations like dilatation and remodeling. On the other hand, it is possible that MRI does not have the necessary sensitivity to detect the iron deposition in the thin atrial myocardium, and the T2* measurements are limited by partial volume effect [21,22]. Another study confirmed the predictive value of T2* for arrhythmia incidence in β-thalassemia patients, also focusing on compliance to iron chelation therapy. Low compliance with chelation was highly predictive of arrhythmias, indicating the importance of adherence to therapy [33].

Since the atria are involved early in β-thalassemia, the atrial function and its role in the pathophysiology of AF have been evaluated by several studies. Russo et al. studied the relationship between an electrocardiographic parameter, the P wave dispersion, and the incidence of AF. The P wave dispersion is the difference between the maximum P wave duration and the minimum P wave duration in the same patient and it represents an indicator of intra-atrial conduction heterogeneity. β-thalassemic patients with preserved systolic and diastolic function, compared to healthy controls, showed significantly higher values of P wave duration and P wave dispersion. Furthermore, P wave dispersion was also inversely related with the iron deposition assessed by MRI [6]. This finding supports the hypothesis that iron overload toxicity per se influences the propagation of sinus impulses and atrial conduction time earlier than affecting mechanical function.

A first, small study published in 2009 showed that thalassemic patients had significantly higher values of left ventricular and left atrial dimensions than healthy controls [34]. In 2014, Kostopolou et al. published the first study that systematically assessed left atrial mechanics and neurohormonal function in patients with β-thalassemia and evaluated their significance as early indexes of myocardial damage. The study prospectively enrolled patients with β-thalassemia and no heart failure, preserved left ventricular ejection fraction and sinus rhythm. Patients with permanent AF were excluded. The E/E’ ratio, which is one of the best echocardiographic indexes of left ventricular diastolic dysfunction, normally increases with age because of the increasing stiffness and lowering compliance of the left ventricle. In β-thalassemic patients, the increase in E/E’ ratio occurred two decades earlier than in healthy controls. Moreover, the development of left ventricular diastolic dysfunction was associated with the impairment of both passive and active atrial mechanical function. Conversely, in controls, depression in passive atrial function was compensated by an increase in atrial emptying resulting in low passive/active atrial function ratios and a later appearance of diastolic dysfunction [22]. An explanation for this finding was that the thin atrial wall may be affected by both iron overload, which represents an infiltrative disease, and inflammation at early stages of the disease and cannot compensate the volume and pressure increases.

**Figure 2:**
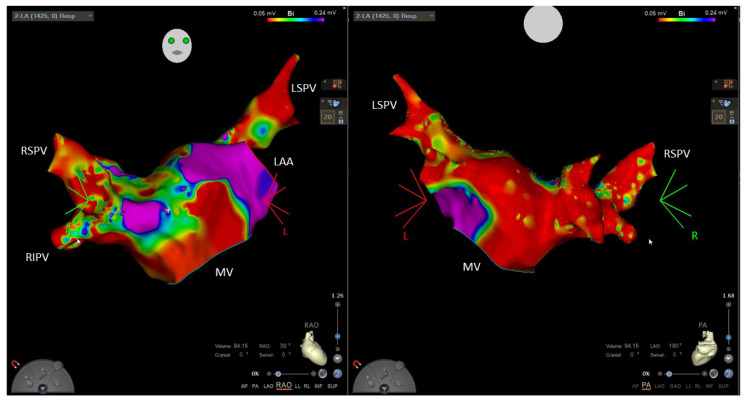
Electroanatomical mapping of left atrium in a patient with β-thalassemia and atrial fibrillation. Panel on the left: right anterior oblique view. Panel on the right: posteroanterior view. Colors indicate electrical potentials. The red color indicates regions with low voltages (<0.05 mV) while purple color indicates normal voltages (>0.24 mV). Of note, a large part of the left atrium shows low electrical potentials, consistent with fibrosis, while normal voltages, identifying healthy muscular walls represent only a small, patchy part of the atrium. MV: mitral valve; LAA: left atrial appendage; LSPV: left superior pulmonary vein; RSPV: right superior pulmonary vein; RIPV: right inferior pulmonary vein.

Thalassemic patients, compared to healthy controls, showed higher levels of natriuretic peptides (NT-proBNP and proANP) with a significant increase after the third decade of life, while in controls, these peptides gradually increase, with a peak value after the fifth decade. Natriuretic peptides are increased in all stages of the disease even in the absence of evident left ventricular dysfunction. NT-proBNP values related with age, iron deposition, left ventricular dimensions and E/E’ ratio [22]. These findings suggest that the atrial dysfunction occur early before left ventricular diastolic and systolic dysfunction, representing a substrate for AF. The atrial wall undergoes fibrosis and remodeling as expression of structural damage (Figure 2).

Another non-invasive indicator of atrial conduction heterogeneity associated with an increased incidence of AF is atrial electromechanical delay (AEMD) [35,36]. AEMD is an echocardiographic index, evaluated with Tissue Doppler technique, consisting of the time interval between the onset of P wave on surface ECG and the beginning of A wave at echo Doppler (PA). It can be obtained either from the lateral mitral annulus (lateral PA), septal mitral annulus (septal PA), or right ventricular tricuspid annulus (right ventricular PA). The difference between these points may reflect the mechanical delay between them. In an observational prospective case–control study, the intra-left AEMD (the difference between lateral and septal PA) and inter-AEMD (the difference between lateral PA and right ventricle PA) were significantly higher in β-thalassemic patients than in healthy controls and were independently related to the incidence of AF [36].

Furthermore, β-thalassemic patients often have an autonomous nervous system dysfunction, which may contribute to the arrhythmogenic substrate. Heart rate variability (HRV), which is an important index of function and balance of the autonomic nervous system, is known to be lower in β-thalassemic patients compared to normal subjects, and it often precedes the signs of cardiac dysfunction. In a study of Yetimakan et al., all HRV parameters were reduced in a group of young thalassemic patients, while no signs of systolic or diastolic dysfunction were detected [37,38]. Of note, autonomic nervous dysfunction may in part be related to diabetes, which frequently affects thalassemic patients because of endocrinopathy directly related to the underlying disease and iron overload [1].

In conclusion, AF in β-thalassemic patients is the result of a multifactorial complex of anemia, transfusions, iron overload, volume overload, hyperkinetic circulation, high diastolic pressure, atrial hemosiderosis, oxygen free radicals, oxidative stress, inflammation, tissue hypoxia, and accelerated vascular aging.

## 4. How to Identify Patients at Risk for Atrial Fibrillation

The main known risk factors for AF in β-thalassemic patients are: iron overload, age, diabetes, left ventricular diastolic dysfunction, electrical and mechanical atrial dysfunction, and natriuretic peptides [22,39].

As indicated above, patients with T2* < 6 ms detected at cardiac MRI have a relative risk of 8.79 (95% CI 4.03–19.2) for arrhythmias at 1 year [21]. However, thalassemic patients frequently develop AF even in the absence of iron deposition at MRI.

Age and diabetes mellitus are strongly associated with AF even in the general population [18]. In patients with β-thalassemia, this association is confirmed [39]. Of note, the risk for developing supraventricular arrhythmias become significant starting from the age of 40, indicating that the occurrence of arrhythmias in thalassemia is far earlier than in the general population [39].

As described before, left ventricular diastolic dysfunction is involved in the development of AF. β-thalassemic patients with a high E/E’ ratio are at higher risk of paroxysmal AF than thalassemic patients with normal E/E’ ratio [22].

Evaluation of atrial function may help to identify thalassemic patients at high risk for AF even in the absence of diastolic and systolic dysfunction. At surface ECG, P wave dispersion >35.5 ms showed a sensitivity of 90% and a specificity of 85%, while maximum P wave duration >111 ms had a sensitivity of 80% and a specificity of 87% [40]. At echo Doppler, AEMD is associated with AF with a cut-off value of 40.1 ms for intra-left AEMD and a cut-off value of 44.8 ms for inter-AEMD, with a sensitivity of 76.2% and 81.2%, respectively, and a specificity of 97.5% and 98.7%, respectively [36]. Atrial dilatation detected at MRI is an independent predictor of supraventricular arrhythmias with a hazard ratio of 4.26 (95% CI 1.54–11.75) [39].

Among β-thalassemic patients, NT-proBNP and proANP are also significantly higher in those at risk for paroxysmal AF compared to those with no arrhythmias [22].

Besides these known risk factors for AF, other common findings may create the substrate for the development of arrythmias. For instance, β-thalassemic patients show a high prevalence of atrial and ventricular extrasystoles, which are known trigger factors for the occurrence of AF. In fact, in several case control studies, β-thalassemic patients have an elevated degree of supraventricular and ventricular extrasystoles detected with ECG-Holter or loop recorder [6,26,41,42]. The frequency of premature atrial contractions was independently associated with the peak of left atrial strain evaluated at speckle tracking echocardiography [43].

The data are still few, but an integrated routine follow-up of β-thalassemic patients including medical history, laboratory tests, ECG recordings, and echocardiographic evaluation should help the physician to recognize patients at high risk of developing AF. Some authors have suggested that these patients should be periodically evaluated with ECG-Holter monitoring for early identification of AF and the commencement of an appropriate treatment [40].

## 5. Clinical Features

Unfortunately, no studies in the literature have specifically evaluated the characteristics of AF in thalassemic patients compared to the general non-thalassemic population. What appears to be evident is that AF in thalassemic patients begins at a younger age [11]. The prevalent form is paroxysmal, while a permanent pattern is less frequent. Of note, the thalassemic patient is much more symptomatic for AF than the non-thalassemic. Such patients, in whom anemia already determines the presence of a hyperdynamic circulation, tend to be warning for palpitations and irregular heartbeat, which are poorly tolerated. As a consequence, arrhythmic recurrences are generally poorly accepted, leading to numerous accesses to the emergency department [16].

## 6. Treatment Options

While several treatments of AF are available and have been extensively studied in the general population, their application in the setting of β-thalassemia is largely unexplored. The peculiarities of thalassemic patients are so many that standard treatments are not automatically translatable in their context. In the absence of specific studies, recommendations for this specific population are no different from that of the general population but there are some fundamental factors that must be taken into consideration (Table 1).

### 6.1. Rhythm Control Drugs

Considering the young age at which thalassemic patients develop AF, obtaining an effective rhythm control should be tried for as long as possible.

First of all, it must be emphasized that, in these patients, iron overload represents the main trigger for the development of arrhythmias. Therefore, if an accumulation of iron in the body is documented, chelation therapy is the best antiarrhythmic drug, and should be considered before any other alternative. Iron chelators can reduce tissue iron levels, preventing iron accumulation, and neutralizing toxic iron pools. Nowadays three iron chelators are commercially available:

- Deferoxamine: this was the first iron chelator introduced in clinical practice. It has a short plasma life and is not absorbed in the gastrointestinal tract, so it must be administered parenterally. Deferoxamine can also be administered in continuous intravenous infusion when intensive chelation is needed [44].
- Deferiprone: is absorbed by the upper gastrointestinal tract so it can be given orally. It may lead to several adverse effects such as gastrointestinal symptoms, arthropathy and agranulocytosis [45].
- Deferasirox: can be given orally once a day. It has a good safety profile, and the main adverse effects are gastrointestinal and renal, which are multiple though rare [46].

From 2000 to 2015, four clinical studies have tested the efficacy of different regimens of these three drugs in managing atrial arrhythmias in patients with iron overload. Even if the number of patients included was low, chelation therapy was effective in regression of arrhythmic disorders in almost all cases [47,48,49,50].

It is known that one of the mechanisms by which iron accumulation causes AF is increased oxidative stress. Therefore, few animal studies have tested the effectiveness of a combination therapy with the addition of antioxidant drugs (N-acetylcysteine, vitamin C, acetaminophen) to chelation therapy to improve cardiac outcome, yielding promising results [15,51].

When the use of antiarrhythmic drugs is necessary, the indications do not differ from those in force for the general population. However, in choosing the best medicine, the particular characteristics of this delicate population must be taken into consideration, and it is often difficult to find a drug that is both effective and well tolerated by the thalassemic patient in the short and long term. Due to the possible interactions with other drugs in this population with multiple comorbidities, caution is required in the use of those drugs that more frequently can determine a proarrhythmic effect or that are contraindicated in patients with underlying heart disease (e.g., flecainide, propafenone, sotalol) [16,52,53]. 

Amiodarone is often both effective in the control of arrhythmic recurrences and safe in the short term. However, its multiple adverse effects when taken chronically, associated with the frequent coexistence of organ damage from iron accumulation, suggest avoiding prolonged use. Long-term therapy, in fact, is often complicated by thyroid and hepatic dysfunction, as these organs are also targets of iron-mediated damage. As a consequence, amiodarone use is usually limited to a duration of a few months while waiting for the chelation therapy to be effective.

### 6.2. Ablation

Catheter ablation has been shown to be a safe and effective option for rhythm control in patients with AF. Current international guidelines recommend ablation for patients with symptomatic AF, both paroxysmal and persistent, who have failed an antiarrhythmic drug therapy (class I of evidence). However, they also add that ablation may be used in selected patients even before a trial of antiarrhythmic drugs (class IIa) [18]. The success rate in controlling arrhythmic recurrences varies from 70 to 80% in the general population [54,55]. However, there are no studies evaluating the efficacy of catheter ablation in patients with β-thalassemia. Catheter ablation success rate in the general population should not be considered applicable for the thalassemic population, in which the pathophysiological mechanisms underlying the arrhythmias are different. Although thalassemic patients are often young (which is usually a prognostic factor of procedure efficacy), they frequently already have dilated atria with large and “patchy” areas of low voltage and fibrosis that make the success of the procedure more difficult to achieve (Figure 2) [16]. However, given the lack of current evidence, patients with β-thalassemia should not be denied the opportunity to benefit from this treatment (which, indeed, should be considered at an early stage, before the atria undergo extensive remodeling). Therefore, at present, the indications for patients with β-thalassemia do not differ from those in valid in the general population, although experts recommend reserving catheter ablation for those patients in whom iron overload has been effectively resolved, after documentation with cardiac MRI [56].

### 6.3. Rate Control

Rhythm control strategy should be preferred in symptomatic patients due to paroxysmal pattern of AF and the presence of a hyperdynamic circulation. However, when this cannot be pursued, in the case of permanent AF or temporarily after the start of chelation therapy, a rate control strategy is necessary. There is no evidence in the literature which suggest the use of specific bradycarding therapies in thalassemia patients. Both β-blockers and non-dihydropyridine calcium channel blockers can be used as the first-choice treatment in patients with preserved ventricular systolic function. Caution should be used in patients with history of heart failure. In these cases, the use of digoxin should be considered [16].

### 6.4. Anticoagulation

The incidence of stroke in the thalassemic population is higher than in the general population, with reported rates of 0.25–0.46% [23,24,25,57]. The presence of a chronic hypercoagulability state has been well documented in patients with β-thalassemia. This seems to be caused by numerous factors including hemoglobinopathy, iron overload, splenectomy, and hemolysis [23,56].

Given the lack of evidence, there are no particular indications on the management of anticoagulant therapy in case of AF in patients with β-thalassemia compared to the general population. In fact, the scores that are actually used in the general population to decide the best antithrombotic strategy are extrapolated from a non-thalassemic population, and have not been validated in patients with hemoglobinopathies. However, hypercoagulability and increased thromboembolic risk in these patients must be taken into account. In the opinion of experts, chronic use of anticoagulant therapy should be considered early on in any case, regardless of the CHA_2_DS_2_-VASc score, except in those cases in which the episodes are very rare and are of short duration, and are compatible with the individual bleeding risk [16,25,56]. However, it should not be forgotten that anemia, and consequently bleeding, is a serious problem in patients suffering from this hemoglobinopathy. Therefore, a careful evaluation must always be made comparing ischemic and hemorrhagic risk on an individual patient basis.

Regarding the best anticoagulation strategy, the most used drug is warfarin, which still represents the standard of care for thromboembolic prevention [16]. The major studies that have tested the safety and efficacy of direct oral anticoagulants (DOACs) have not been conducted in specific populations of patients with hemoglobinopathies. However, it is evident that DOACs are more manageable than warfarin and have a more favorable safety profile [58,59]. Only one study tested the effectiveness of rivaroxaban in a very small group of patients with β-thalassemia and AF. In five patients, the direct inhibitor of factor X was administered, while in three patients, warfarin was prescribed; during the follow-up none of the eight patients suffered from any event of either ischemic or hemorrhagic type [60]. Further data are therefore necessary in order to assess the non-inferiority of DOACs respect to vitamin K inhibitors in patients with hemoglobinopathies. 

## 7. Conclusions

Nowadays, AF is a frequent complication of β-thalassemia, and its management is part of daily clinical practice. Unfortunately, evidence is sparse, and ad hoc studies in this specific setting are lacking. The evidence and recommendations valid for the general population are difficult to apply to patients with β-thalassemia. Patients with β-thalassemia show higher prevalence of AF, higher thromboembolic risk, greater severity of symptoms, and higher rate of complications. Identification of patients at risk, careful evaluation of thromboembolic risk and early rhythm control strategy with drugs or ablation are the criteria that should guide the clinical management.

## Figures and Tables

**Table 1:** Therapeutic options for the management of atrial fibrillation in patients with β-thalassemia. HF: heart failure; INR: international normalized ratio; DOACs: direct oral anticoagulants.

	Option	Pros	Cons	Caution in Thalassemia
**Rhythm control strategy**	**Chelation therapy**	Effective in preventing both arrhythmic recurrences and iron overload		First line therapy in transfusion-dependent patients
	**Amiodarone**	Effective Safe in the short term	Multiple adverse effects in the long term	Frequent coexistence of organ damage (thyroid, liver, skin)
	**Other antiarrhythmic drugs (flecainide, propafenone, sotalol)**	Less side effects in the long term	Drug interactions Contraindicated if underlying HF	Possible proarrhythmic effect in patients with iron overload cardiopathy
	**Catheter ablation**	Avoiding side effects of antiarrhythmic drugs	Invasive procedure	Atrial structural cardiopathy limiting efficacy
**Rate control strategy**	**β-blockers**	Effective in reducing symptoms when rhythm control is not possible Indicated also for HF	Negative chronotropic and inotropic effect	Bradycardia may be poorly tolerated
	**Calcium channel blockers (verapamil, diltiazem)**	Effective in reducing symptoms when rhythm control is not possible	Contraindicated in HF with reduced ejection fraction	Possible coexistence of HF Bradycardia may be poorly tolerated
	**Digoxin**	Second line therapy when β-blockers or calcium channel blockers are not tolerated	Small therapeutic window Possibility of overdose	Multiple drug interactions
**Anticoagulation**	**Warfarin**	Frequent monitoring of coagulation state (INR)	Frequent blood test Labile INR values Less manageable and safe than DOACs	Higher hemorrhagic risk
	**DOACs (apixaban, dabigatran, edoxaban, rivaroxaban)**	More manageable and safe than warfarin		Higher hemorrhagic risk

## Data Availability

Not applicable.
